# T cell autoimmunity and immune regulation to desmoglein 3, a pemphigus autoantigen

**DOI:** 10.1111/1346-8138.16663

**Published:** 2022-12-20

**Authors:** Hayato Takahashi, Hisato Iriki, Yasuhiko Asahina

**Affiliations:** ^1^ Department of Dermatology Keio University School of Medicine Tokyo Japan

**Keywords:** desmoglein, human leukocyte antigen, pemphigus, T cells, tolerance

## Abstract

Pemphigus is a life‐threatening autoimmune bullous disease mediated by anti‐desmoglein IgG autoantibodies. Pemphigus is mainly classified into three subtypes: pemphigus vulgaris, pemphigus foliaceus, and paraneoplastic pemphigus. The pathogenicity of autoantibodies has been extensively studied. Anti‐human CD20 antibody therapy targeting B cells emerged as a more effective treatment option compared to conventional therapy for patients with an intractable disease. On the other hand, autoreactive T cells are considered to be involved in the pathogenesis based on the test results of human leukocyte antigen association, autoreactive T cell detection, and cytokine profile analysis. Research on the role of T cells in pemphigus has continued to progress, including that on T follicular helper cells, which initiate molecular mechanisms involved in antibody production in B cells. Autoreactive T cell research in mice has highlighted the crucial roles of cellular autoimmunity and improved the understanding of its pathogenesis, especially in paraneoplastic pemphigus. The mouse research has helped elucidate novel regulatory mechanisms of autoreactive T cells, such as thymic tolerance to desmoglein 3 and the essential roles of regulatory T cells, Langerhans cells, and other molecules in peripheral tissues. This review focuses on the immunological aspects of autoreactive T cells in pemphigus by providing detailed information on various related topics.

## 1 INTRODUCTION

Pemphigus is a life‐threatening autoimmune bullous disease mediated by anti‐desmoglein IgG autoantibodies. Pemphigus is mainly classified into three subtypes, pemphigus vulgaris (PV), pemphigus foliaceus (PF), and paraneoplastic pemphigus (PNP).^1^

PV and PF are the classical presentations of pemphigus. PV develops mucosal lesions with or without cutaneous lesions and histologically suprabasilar acantholysis. PF develops cutaneous lesions with acantholytic blisters beneath the stratum corneum, an upper layer of the epidermis. In PF, anti‐desmoglein (Dsg) 1 autoantibodies are responsible for inducing acantholysis, as Dsg1 is the primary adhesive molecule of desmosomes in the upper epidermis.^2^, ^3^, ^4^ In contrast, desmoglein 3 (Dsg3) is predominantly expressed in the epithelium of the mucous membrane. Both Dsg1 and 3 are expressed in the lower layer of the skin epidermis. Therefore, anti‐Dsg3 antibodies cause blisters only in the mucosal membrane, such as the oral mucosa, but not in the skin, presenting clinically as mucosal‐dominant PV, since Dsg1 compensates for the loss of Dsg3 function by autoantibodies in the skin.^2^ In the scenario where both anti‐Dsg1 and anti‐Dsg3 autoantibodies are induced in the patients, blisters occur in the lower layer of the epidermis and the mucous membrane, presenting clinically as a mucocutaneous type of PV.

PNP is a more recently described subtype of the disease.^5^ PNP patients show a variety of eruptions, including erythema multiforme‐like eruptions and maculopapular eruptions, along with blisters. In particular, mucosal lesions are often intractable to standard treatments and result in a poor quality of life. Histologically, interface dermatitis can be observed as well as acantholysis, unlike with PV and PF, although anti‐Dsg3 antibodies in PNP patients were also shown to be responsible for inducing acantholytic blisters.^6^

In all three subtypes, anti‐Dsg IgG autoantibodies are responsible for the disease symptoms. Research on pathogenic antibodies has helped develop therapeutic approaches targeting autoreactive B cells or autoantibodies. The recently introduced anti‐CD20 therapy^7^ is a more effective option for intractable cases compared with conventional therapy. Its therapeutic use against pemphigus has dramatically improved patient prognosis in clinical practice. However, several fundamental questions remain unclear, including why pemphigus occurs and how dysregulated immunological conditions can be restored in pemphigus. This review focuses on the research investigating T cell autoimmunity and immune regulation induced by Dsg3.

## 2 T CELL AUTOIMMUNE RESPONSE IN PEMPHIGUS

### 2.1 Evidence supporting T cell involvement in pemphigus pathogenesis

CD4^+^ helper T cells are necessary for efficient antibody production during acquired immunity. Along with the interaction between cognate T cells and B cells in the germinal center, immunoglobulin genes undergo class‐switch recombination and somatic hypermutation in complementarity‐determining regions. Evidence of CD4^+^ T cell involvement in pemphigus pathogenesis has been reported. The predominant subclass of anti‐Dsg3 IgG was IgG4 in pemphigus, suggesting T cell‐dependent isotype switching.^8^, ^9^ Also, T cell‐dependent somatic hypermutations were observed in complementarity‐determining regions of some anti‐Dsg3 IgG genes, although some genes encoding pathogenic antibodies underwent only a few mutations.^10^, ^11^

**FIGURE 1:**
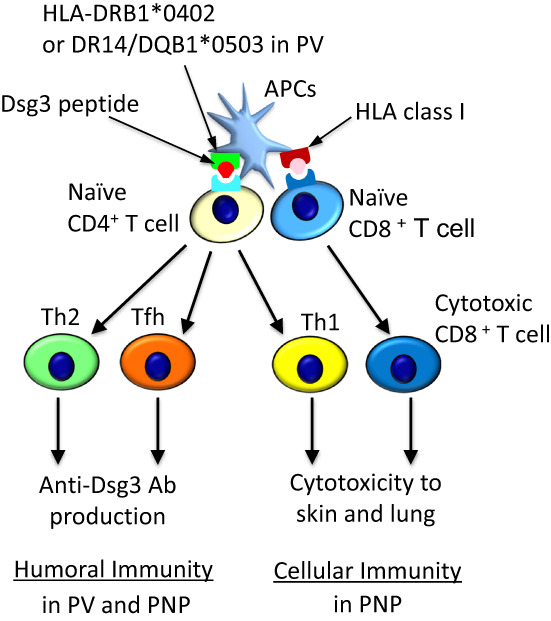
Schematic overview of humoral and cellular immunity in pemphigus vulgaris and paraneoplastic pemphigus. Ab, antibody; APC, antigen presenting cells. HLA, human leukocyte antigen.

HLA allele association with anti‐Dsg antibody production is evidence for T cell involvement in the disease because HLA molecules present antigenic peptides to T cells. The peptide repertoire that binds HLA molecules depends on the type of HLA allele because the molecular structure of the groove where the peptide fits differs among the alleles. In pemphigus, it is a reasonable assumption that HLAs that bind with specific Dsg3 peptides are involved in pathogenesis (Figure 1). A strong association of several alleles of HLA class II genes was previously reported.^1^ HLA‐DRB1*0402 was reported to be strongly associated with PV in Ashkenazi Jewish patients, while HLA‐DRB1*14 and HLA‐DQB1*0503 were associated with non‐Jewish European and Asian patients. Since HLA‐DR14 is associated with HLA‐DQB1*0503 owing to linkage disequilibrium, it is difficult to determine which allele is primarily responsible for anti‐Dsg3 antibody production. The strong association of certain HLA class II alleles with anti‐Dsg antibody production suggests the involvement of autoreactive CD4^+^ helper T cells in pemphigus pathogenesis.

Several classic HLA class I molecules are reportedly associated with the disease,^12^ but the results are not easy to interpret since CD8^+^ T cells that interact with HLA class I molecules do not directly relate to autoantibody production. One study demonstrated that *CD8* ^−/−^ neonatal mice were resistant to blister formation after the passive transfer of PV‐patient‐derived IgG.^13^ However, the detailed roles of CD8^+^ T cells in autoantibody‐mediated blister formation remain unclear.

The study enrolled 13 Caucasian patients with PNP and revealed that HLA‐DRB1*03 was associated with the disease. HLA‐DR4 and DR14, which were significantly associated with PV and PF, were not significantly observed.^14^ Another study with 19 Chinese patients with PNP demonstrated that HLA‐Cw*14 was significantly associated, but HLA‐DRB1*03 associated with Caucasian patients was not.^15^ Since PNP is a rare form of pemphigus in which humoral and cellular immunity is observed, the sample sizes of these studies were relatively small and larger sample sizes are required for better conclusions. However, the immunological background of PNP appears different from that of PV and PF. In particular, the association of HLA class I molecules with PNP observed in Chinese patients might be interesting when considering the contribution of CD8^+^ T cells to disease pathogenesis, especially cellular autoimmunity in PNP, as discussed below.

### 2.2 HLA class II restriction to autoreactive T cells in pemphigus

T cell autoreactivity was investigated earlier in three separate studies. The first study predicted the Dsg3 peptide that fits pockets in a groove of the PV‐related HLA molecule based on the specific amino acid sequence of HLA‐DRB1*0402.^16^ Proliferative responses of T cells to Dsg3 (190–204 amino acids (aa)), Dsg3 (206–220 aa), Dsg3 (251–265 aa), and Dsg3 (762–786 aa) peptides were observed in the active phase of patients with HLA‐DRB1*0402. A T cell clone specific to Dsg3 (190–204 aa) was established, and it was experimentally demonstrated that the Dsg3 (190–204) peptide was specifically presented by HLA‐DRB1*0402 but not by other HLA‐DRB1*04 alleles.

The second study found T cells from PV patients whose disease activity was not detailed proliferating to Dsg3 (145–192 aa), Dsg3 (240–303 aa), or Dsg3 (570–614 aa) proteins.^17^ One T cell clone derived from PV patients with HLA‐DRB1*0701/1401 and DQB1*0201/0503 responded to Dsg3 (145–192 aa). An anti‐HLA‐DR antibody inhibited this response, but not an anti‐DQ antibody. Hence, HLA‐DRB1*1401 rather than HLA‐DQB1*0503 was the primary allele responsible for PV development. Interestingly, this clone responded to the Dsg3 peptide under antigen presentation by antigen‐presenting cells (APCs) expressing either HLA‐DRB1* 1401 or HLA‐DRB1*0402. This implied that both alleles presented the Dsg3 peptide to T cells. Regarding HLA‐DQB1*0503 restriction in T cell reactivity to Dsg3, one study demonstrated that the in vitro reactivity of a Dsg3‐reactive T cell clone derived from a patient with HLA‐DQB1*0503 was suppressed by an anti‐HLA‐DQ antibody but not an anti‐HLA‐DR antibody,^18^ suggesting HLA‐DQB1*0503 as the responsible allele.

The third early study enrolled PV patients and healthy controls.^19^ T cell responses to Dsg3 protein were observed not only in patients but also in healthy controls. T cell lines derived from patients with HLA‐DRB1*0402/1104 and DQB1*0301/0302 showed a proliferative response to Dsg3 presented by HLA‐DRB1*0402‐expressing APCs. In addition, T‐cell clones from HLA‐DR11^+^/DQB1*0301^+^ patients and healthy controls responded to the Dsg3 protein in an HLA‐DQB1*0301‐restricted manner. HLA‐DQB1*0503 is a rare allele strongly associated with PV and differs from the common allele DQB1*0501 only in a valine to aspartic acid substitution at position β57. As DQB1*0301 also has this aspartic acid,^19^ and crucial roles of the amino acid at position DQβ57 in the binding of antigenic peptides have been reported,^20^ the structural similarity between DQB1*0503 and DQB1*0301 was attributed to this observation in the study that HLA‐DQB1*0301, the allele rarely seen in PV patients, was the restricted allele for Dsg3 presentation to T cells.

These combined results suggest that HLA‐DRB1*0402 is primarily responsible for Dsg3 peptide presentation to CD4^+^ T cells in DRB1*0402‐bearing patients with PV. However, for other PV patients, HLA class II restriction, including HLA‐DR14 or DQB1*0503 restriction for Dsg3 presentation, remained undetermined and might need to be elucidated for each case of patients and peptides (Figure 1).

### 2.3 Cytokine profiles in autoreactive T cells in pemphigus

The Th1/Th2 imbalance often explains the pathogenesis of immunological diseases. Extensive research has investigated the serum cytokine concentrations in patients with pemphigus. Increased levels of serum IL‐4 and IL‐10 and decreased levels of serum IL‐2 and IFN‐γ were reported, suggesting Th2 deviation.^21^ While serum IL‐6 was reportedly elevated,^22^ other studies reported no significant difference in serum IL‐6 in PV.^23^ Cytokines are produced by various types of cells in the body, and serum cytokine concentration changes depending on the cytokine and disease condition analyzed. Although there are several controversial results on the cytokine profile in pemphigus, one systematic review of cytokine study in pemphigus reported increased levels of TNF‐α, TGF‐β, IL‐8, IL‐10, IL‐12, IL‐17, and IL‐21 and decreased levels of IL‐2 and IL‐23.^24^ However, rather than knowing the total cytokine concentration in the serum, elucidating cytokine profiles in each cell type would be more helpful in understanding the pathogenesis, since cytokine are usually thought to function immediately after secretion from immune cells in the microenvironment, where immune reactions occur, not after diffusion to the whole body.

**FIGURE 2:**
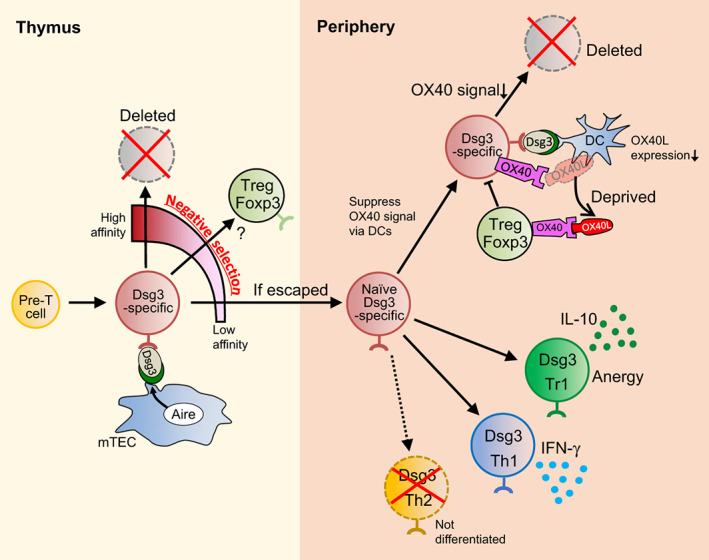
Schematic overview of central and peripheral tolerance against Dsg3‐specific T cells. When pre‐T cells express Dsg3‐specific TCR to be Dsg3‐specific T cells, the cells receive Dsg3‐antigen presentation from mTECs which express Dsg3 in an Aire‐dependent manner as negative selection in central tolerance mechanisms. Based on the TCR affinity to Dsg3, the cells usually either undergo deletion or might differentiate into Treg. If the cells escape the central tolerance, the cells distribute in the periphery, but additionally undergo peripheral tolerance mechanisms such as IL‐10‐producing Tr‐1 differentiation, and being deviated in Th1 with Th2 differentiation prohibited, as well as deletion due to reduced OX40 signal directed by Tregs; Tregs deprive OX40L of antigen‐presenting DCs.

Several studies have investigated cytokine production by autoreactive T cells. The group which conducted a comprehensive analysis of cytokine production from Dsg3‐reactive T cells in humans^18^ divided patients into three disease phases, acute onset phase, chronically active phase, and remittent phase. IL‐4‐secreting Dsg3‐resctive Th2 cells were detected in all three phases of the disease but not in healthy controls (Figure 1). IFN‐γ‐secreting Dsg3‐reactive Th1 cells were significantly increased in the chronic active phase. In contrast, in healthy individuals who carried PV‐related HLA class II alleles, HLA‐DRB1*0402, DQB1*0503, or both, only Dsg3‐reactive Th1 cells were detected (Figure 2), whereas neither Th1 nor Th2 responses were detected in healthy individuals who carried PV‐unrelated HLA class II alleles. These Th1 or Th2 cells were isolated as T cell clones after long‐term in vitro culture, but the cytokine profile of the T cell clones did not change. Consistently, IL‐4 production from Dsg3‐reactive T cell clones has also been observed in other studies.^17^ These results indicated that the Th2 phenotype seems crucial for anti‐Dsg3 antibody production, and immunological responses against Dsg3 were quite different between patients and healthy individuals. Moreover, the breakdown of specific regulatory mechanisms that prevent Dsg3‐reactive Th2 responses may be involved in PV pathogenesis.

### 2.4 T follicular helper cells in pemphigus

For efficient antibody production from B cells, cognate T cell–B cell interaction is a critical step in which CD40‐CD40L interaction drives IgM to IgG isotype switch and somatic hypermutation for affinity maturation of immunoglobulin.^25^ This interaction occurs in the germinal center of the secondary lymphoid organs, and CD4^+^ helper T cells specialized for this process are called T follicular helper (Tfh) cells.^26^ Since pemphigus is mediated by anti‐Dsg IgG autoantibodies, it is reasonable to think that Tfh cells are involved in the pathogenesis of the disease (Figure 1). As summarized below, four recent studies have provided important evidence on Tfh involvement in pemphigus pathogenesis.

Tfh cells reside in lymph nodes but circulating CD4^+^CXCR5^+^ Tfh‐like cells that support antibody production are also detected in peripheral blood.^27^ Circulating PD‐1^+^CXCR5^+^ Tfh‐like cells showed similar levels in PV and healthy controls, and only a proportion of inducible costimulator (ICOS)^+^ Tfh‐like cells, but not ICOS^−^ Tfh‐like cells, positively correlated with anti‐Dsg3 antibody titers.^28^ In addition, ICOS^+^ Tfh‐like cells were further divided into Th1, Th2, and Th17 cells based on their chemokine receptor expression (Th1: CXCR3^+^CCR6^−^, Th2: CXCR3^−^CCR6^−^, Th17: CXCR3^−^CCR6^+^).^29^ The proportions of Th2 and Th17 subpopulations were increased within ICOS^+^ Tfh‐like cells, whereas only the Th2 subpopulation within ICOS^+^ Tfh‐like cells correlated with anti‐Dsg3 antibody titers.^28^ Consistent with this result, Dsg3‐specific Tfh‐like cells were decreased in proportion after rituximab therapy in pemphigus patients.^30^

In another study, whole transcriptome analysis of lesional skin samples revealed elevated *IL4* in PF, but not PV,^31^ while *IFNG*, *IL10*, *IL1A*, and Th17‐related cytokines including *IL17A*, *IL23A*, *IL21*, *IL19*, and *IL24* were elevated in PV and PF. Chemokine receptor expression was used to identify Th1, Th17, and Th2 cells, similar to the aforementioned study. The proportion of circulating Th17 cells and Th17 subpopulation within CXCR5^+^ Tfh‐like cells was positively correlated with Dsg3‐specific B cell number. In particular, the Th17 subpopulation within Tfh‐like cells contained a higher frequency of the Dsg3‐reactive population, identified as CD154‐expressing cells, on stimulation with recombinant Dsg3 (rDsg3) protein in the active phase of PV than in the remission phase of PV and healthy controls. Also, the Th17 subpopulation within Tfh‐like cells efficiently induces anti‐Dsg antibody production in vitro.

Another study showed the critical roles of skin‐resident memory T cells with a Tfh‐like phenotype in local autoantibody production.^32^ Resident memory T cells (Trm) are a subset of memory T cells that reside in various tissues without circulation and can rapidly initiate an immune response against invading pathogens at re‐infection.^33^ The study demonstrated that skin‐infiltrating CD4^+^CD69^+^CCR7^−^ Trm was more prevalent in pemphigus patients than in healthy controls and was positively correlated with the pemphigus disease area index. Transcriptome analysis revealed that Tfh‐related genes, such as *ICOS*, *TNFRSF4* (encoding OX40), *CD40LG*, *IL21*, and *IRF4*, were elevated in skin‐infiltrating CD4^+^ Trm. IL‐17A and IL‐21 production was confirmed in the Trm at protein levels. In vitro culture of lymphocytes isolated from pemphigus skin lesions resulted in anti‐Dsg3 antibody production, significantly decreased by Trm depletion or IL‐21 blockade in the culture system.

Together, these results support the hypothesis that Tfh is involved in anti‐Dsg antibody production in pemphigus. One hurdle of human studies is the difficulty of clarifying whether the analyzed T cells induce pathogenic antibody production and disease phenotype in vivo. Mouse studies complement what is not known from human studies.

## 3 ANIMAL MODEL OF PEMPHIGUS

### 3.1 Passive transfer model of pemphigus

Several animal models of pemphigus have been generated to elucidate the disease mechanism of pemphigus and evaluate new therapeutic strategies in preclinical studies. A passive transfer model was originally developed to assess the pathogenicity of the antibodies of interest. For example, IgG fractions prepared from pemphigus patients' sera, monoclonal antibodies, or single‐chain variable fragments from phage display can be used for the model.^6^, ^34^, ^35^, ^36^ When pathogenic antibodies are injected intraperitoneally or subcutaneously into neonatal mice, acantholytic blisters develop in the skin 12–18 h after the injection. Hybridoma cells that produce pathogenic anti‐Dsg3 antibodies can be adoptively transferred into immunodeficient mice as a modified version of the passive transfer model^34^ and used to evaluate new therapeutic strategies, such as chimeric autoantibody receptor‐expressing T cell therapy.^37^

### 3.2 Active disease model of pemphigus

To observe and analyze the roles of Dsg3‐specific T cells in animal models, the model must be generated by including a Dsg3‐specific T cell reaction that cannot be observed in the passive transfer model generated by antibodies or B‐cell hybridoma cells. The representative model for this purpose is the active disease model in which splenocytes isolated from *Dsg3* ^−/−^ mice immunized with rDsg3 protein were adoptively transferred into *Rag2* ^−/−^ mice that lack T and B cells but normally express Dsg3 in the squamous epithelium, such as the skin.^38^ Since immunological tolerance against Dsg3 is not established in *Dsg3* ^−/−^ mice, T and B cells in *Dsg3* ^−/−^ mice can initiate a Dsg3‐specific immune reaction after immunization with rDsg3 protein and persistently produce anti‐Dsg3 IgG antibodies after adoptive transfer driven by endogenous Dsg3 antigen expressed in *Rag2* ^−/−^ mice. Recipient *Rag2* ^−/−^ mice developed acantholytic blisters in histology and the pemphigus phenotype. This Dsg3‐specific immune reaction was mediated only by a combination of T cells and B cells derived from *Dsg3* ^−/−^ mice but not perfectly achieved when either of the cell populations were derived from wild‐type mice, suggesting the necessity of T cell–B cell interaction and loss of immunological tolerance against Dsg3 in both populations for the development of the pemphigus phenotype in this animal model.^39^

Several Dsg3‐specific T cell clones were established and assessed using this active disease model.^40^ IL‐4 produced from the clones was crucial for inducing anti‐Dsg3 antibody production, demonstrating the importance of the Th2 phenotype of Dsg3‐specific T cells, which was also suggested in a human study as described above. In another study, only ICOS^+^CXCR5^+^PD‐1^+^ Tfh cells were identified as T cell populations capable of inducing anti‐Dsg3 antibody production in the model.^28^ In addition, Dsg3‐specific Tfh cells were detected using I‐A^b^ tetramer with Dsg3 (516–530 aa) peptide highly expressed ICOS, and ICOS blockade reduced anti‐Dsg3 antibody production in the model, demonstrating pivotal roles of Dsg3‐specific ICOS^+^CXCR5^+^PD‐1^+^ Tfh cells in the pemphigus mouse model. Another model using mouse major histocompatibility complex (MHC) class II‐deficient HLA‐DRB1*0402 transgenic mouse helped analyze the interaction between human Dsg3 peptide and pemphigus‐related HLA molecules without obtaining Dsg3‐specific T cell clones.^41^ These results indicate the usefulness of the mouse model to deeply dissect disease mechanisms that are difficult to conduct in human samples.

### 3.3 Animal model of cellular autoimmunity against Dsg3

Dsg3‐reactive T cell clones that induced anti‐Dsg3 antibody production and pemphigus phenotype were originally established from *Dsg3* ^−/−^ mice, where the immunological tolerance mechanism against Dsg3 did not function. One study generated Dsg3‐specific T cell receptor (TCR) transgenic mice (Dsg3H1 mice), in which transgenic Dsg3‐specific CD4^+^ T cells developed in vivo, to understand how autoreactive CD4^+^ T cells specific to physiological epidermal autoantigens were regulated by the immune system.^42^ Dsg3H1 mouse itself showed mild inflammation in the skin. When the transgenic T cells were adoptively transferred into *Rag2* ^−/−^ mice, the T cells directly infiltrated the Dsg3‐expressing epidermis. They attacked keratinocytes, leading to the development of Civatte bodies, liquefaction degeneration, and degenerated keratinocytes. These pathological changes were consistent with interface dermatitis. On the other hand, when Dsg3‐specific CD4^+^ transgenic T cells developed in the absence of Dsg3, they were able to induce anti‐Dsg3 antibody production and acantholysis in histology as well as interface dermatitis after the adoptive transfer into *Rag2* ^−/−^ mice together with *Dsg3* ^−/−^ B cells, demonstrating the potential of Dsg3‐specific T cells to induce both interface dermatitis and acantholysis (Figure 1). This transgenic system helped elucidate several novel immunological findings associated with T cell‐mediated autoimmune responses, such as the essential roles of IFN‐γ‐and the immunoregulatory function of Langerhans cells and cholesterol 25‐hydroxylase expressed in CD4^+^ T cells in interface dermatitis.^42^, ^43^, ^44^

The experimental models for pemphigus discussed here have contributed to our deeper understanding of disease pathogenesis and the potential of new therapeutic strategies.

## 4 PATHOGENIC ROLE OF AUTOREACTIVE T CELLS IN PNP

### 4.1 Clinical and pathological features of PNP

PNP is a distinctive variant of pemphigus associated with neoplasms.^1^, ^45^ PNP is less common than PV and PF, and 84% of the underlying neoplasms are hematologic‐related disorders, of which approximately 40% are non‐Hodgkin lymphomas, 15%–37% correspond to Castleman disease, and 7%–18% correspond to chronic lymphocytic leukemia.^46^, ^47^, ^48^ There are cases of PNP associated with non‐hematological diseases, such as thymoma, adenocarcinoma, and squamous cell carcinoma.^49^, ^50^, ^51^, ^52^

Unlike PV and PF, the cutaneous manifestations of PNP are polymorphic, ranging from pemphigus‐like or pemphigoid‐like blisters and erosions to erythema multiforme‐like targetoid lesions and graft‐versus‐host disease‐like or lichen planus‐like erythematous papules and plaques.^45^, ^53^ Different skin lesions can be present in the same patient. Refractory and recurrent oral mucosal erosions are often observed in patients with PNP. Typically, painful erosions in the buccal mucosa and tongue are presented early in the disease.^54^ Oral lesions are often more extensive and severe than PV, and some cases present with only mucosal involvement.^1^, ^55^, ^56^, ^57^ The histopathological features also vary. Histopathology shows acantholytic blisters, while dermal dense inflammatory infiltrates and Civatte bodies forming pathological changes, such as interface dermatitis, are also observed in graft‐versus‐host disease‐like or lichen planus‐like lesions.^58^ These observations are often seen simultaneously in the same lesion. PNP is a unique skin disease resulting from acantholysis and interface dermatitis.

### 4.2 Humoral autoimmunity in PNP

A wide variety of skin manifestations and histopathological findings of PNP occur because of both humoral and cell‐mediated autoimmunity (Table 1). Autoantibodies against desmogleins are often detected, although some cases occur without anti‐desmoglein antibodies.^59^, ^60^ In addition to anti‐desmoglein antibodies, patients with PNP have multiple antibodies, including antibodies against desmocollin 1–3, α‐2‐macroglobulin‐like‐1 protein, and plakin family members (desmoplakin, epiplakin, periplakin, and envoplakin).^47^, ^61^, ^62^, ^63^ Presence of autoantibodies against envoplakin and periplakin is sensitive to the diagnosis of PNP.^58^ These antibodies are also detectable in patients with erythema multiforme.^64^ The pathogenicity of antibodies against plakin family members that are localized intracellularly is unclear. One study reported that purified anti‐desmoplakin antibody from a patient with PNP induced dissociation of keratinocytes in vitro and caused blisters in vivo when transferred to neonatal mice. A possibility that the contaminated anti‐Dsg3 antibody in the purified antibodies could cause blister formation remains elucidated.^65^ And the potential pathogenicity of monoclonal antibodies against plakin family proteins has not yet been investigated. Clinically, it is often observed that severe mucosal lesions persist even after the Dsg3 antibody becomes undetectable due to treatment, suggesting that cell‐mediated immune responses recognized in pathology and humoral immunity are crucial aspects to consider in the pathogenesis of PNP.

**TABLE 1:** Immunological features of pemphigus vulgaris/foliaceus and paraneoplastic pemphigus

	Humoral immunity	Cellular immunity	Complication in lung
Pemphigus vulgaris and pemphigus foliaceus	Yes, against Dsg3 and Dsg1	No	No
Paraneoplastic pemphigus	Yes, against Dsg1/3, Dsc1‐3, A2ML1, and plakin family members	Yes, antigens have not been identified	Yes, BO

Abbreviations: A2Ml1, α‐2‐macroglobulin‐like‐1; BO, bronchiolitis obliterans; Dsc, desmocollin; Dsg, desmoglein.

### 4.3 Cellular autoimmunity and bronchiolitis obliterans in PNP

The cytotoxicity of autoreactive T cells reacting to epithelial and epidermal cells causes refractory oral lichenoid mucositis and lichen planus‐like eruptions.^1^ Experimentally, transgenic CD4^+^ T cells expressing TCR specific for Dsg3 induced acantholytic blister formation and interface dermatitis when co‐transferred with B cells from *Dsg3* ^−/−^ mice into immunodeficient mice.^42^ This mouse model suggested that cytotoxicity by autoreactive T cells against Dsg3 is responsible for interface dermatitis. In PNP patients, lichenoid dermatitis can emerge before autoantibodies are detected or can be the only symptom.^66^, ^67^ Interface dermatitis may cause exposure of plakin family members to the immune system, followed by the induction of autoantibodies against multiple antigens, which is often explained as epitope spreading.^68^

Bronchiolitis obliterans (BO) is a potentially lethal complication of PNP and another clinical feature that can be mediated by both humoral and cellular autoimmunity. In BO, inflammation and fibrosis of the bronchus result in obstructive changes, clinically presenting with dry cough, dyspnea, and hypoxemia.^69^ BO is caused by infection, inhalational injury, bone‐marrow transplantation, lung or heart‐lung transplantation, drug toxicity, or collagen‐vascular diseases.^69^ BO affects about 30% of patients with PNP and is responsible for the low survival rate of PNP.^70^

Bronchial epithelial tissues from PNP patients with BO revealed acantholysis and deposition of IgG on the cell surfaces.^71^ Mice transferred with polyclonal antibodies specific to epiplakin showed detached epithelial cells in the lung, suggesting a loss of cell–cell adhesion.^72^ These studies indicate that autoantibodies contribute to the pathogenesis of BO.

Cellular immunity mediated by autoreactive CD8^+^ T cells may play essential roles in BO (Figure 1). Dense infiltrates of CD8^+^ T cells are observed in the lung epithelium of BO.^73^ Here, PNP model mice were created by adoptive transfer of splenocytes from *Dsg3* ^−/−^ mice immunized with wild‐type skin grafts to *Rag2* ^−/−^ mice.^74^ They exhibit acantholysis and interface dermatitis with CD4^+^ and CD8^+^ T cell infiltration. The mice also showed pulmonary infiltration with ectopic Dsg3 expression in the lungs. Dsg3 and keratin 5 and 14 were expressed in the pulmonary tissue in which squamous metaplasia was experimentally induced. A lung autopsy sample from a patient with PNP showed squamous metaplasia. These data suggest that specific epidermal autoantigens can be target antigens of autoreactive T cells in PNP, and ectopic expression of the epidermal autoantigen via squamous metaplasia may make the lung susceptible to autoimmune reactions by epidermal antigen‐reactive T cells that normally do not attack the lungs.

PNP is mediated via complex pathogenesis involving humoral and cellular immunity, and the wide range of antibody profiles suggests that the pathogenesis of PNP varies from patient to patient. Elucidating the etiology and properties of autoreactive T cells should help to better understand PNP pathogenesis.

## 5 T CELL TOLERANCE IN PEMPHIGUS

### 5.1 Important immunological tolerance to Dsg3

The development of autoimmune diseases is a negative aspect associated with the high diversity of the immune system to resist various foreign pathogens. To prevent this, a defense mechanism ensures that attacks against self‐antigens do not occur. This mechanism is called immune tolerance, and its breakdown is thought to be the initial step toward the onset of autoimmune diseases.

In animal models, an important immunological tolerance against Dsg3 can be found in developing an active disease model for pemphigus, as described above. Dsg3‐specific immune reactions can only be efficiently obtained when both T and B cells are prepared from *Dsg3* ^−/−^ mice for adoptive transfer. This evidence suggests that immunological tolerance to Dsg3 at the B and T cell levels is a potent mechanism to prevent Dsg3‐specific immune reactions in wild‐type mice and that flaws in any part may induce an autoimmune reaction to Dsg3.^39^

Immune tolerance can be classified into two categories: central and peripheral. Regarding the pemphigus antigen Dsg3, the tolerance mechanisms against Dsg3‐specific T cells in the thymus and periphery are becoming better understood (Figure 2) and are described in detail below.

### 5.2 Thymic central tolerance to Dsg3

The central immune tolerance mechanism operates during T cell development in the thymus.^75^ In the developmental process of T cells, progenitor cells generated in the bone marrow enter the thymus cortex, where they undergo TCR gene rearrangement and express a variety of TCRs.^76^ Only those of these cells that can react moderately to self‐antigens on MHC molecule presented by thymic cortical epithelial cells survive and migrate to the thymic medulla; this step is called positive selection.^77^ Subsequently, central immune tolerance mechanisms efficiently suppress the harmfulness of T cells with TCRs that react to self‐antigens presented by the thymic medullary epithelium^78^; this is negative selection.^77^ In this step, T cells with high affinity to self‐antigens expressed in medullary thymic epithelium cells (mTECs) are eliminated by apoptosis induction, while those with relatively low affinity are forced to differentiate into Tregs and others pass this selection.^79^, ^80^ Self‐antigen presentation by mTECs is mediated by the transcription factors autoimmune regulator (AIRE) or FEZ family zinc finger 2 (FEGF2), which are responsible for the comprehensive thymic expression of self‐antigens that are usually expressed in various peripheral organs in the body.^81^, ^82^ Dsg3 is one of the self‐antigens to be expressed in mTECs under the control of the Aire.^83^ Dsg3‐expression in mTEC is lacking in *Aire*−/− mice, as observed in *Dsg3* ^−/−^ mice (Figure 2).

Direct evidence of central tolerance against Dsg3‐specific T cells was provided in an experiment using Dsg3H1 mice. When Dsg3H1 mice were crossed with *Rag2* ^−/−^ mice, endogenous TCR expression was diminished in the transgenic T cells, therefore the fate of Dsg3‐specific transgenic CD4^+^ T cells can be observed without the influence of endogenous TCRs other than Dsg3‐specific TCR. When bone marrow from Dsg3H1‐*Rag2* ^−/−^ mice was transplanted into WT and *Dsg3* ^−/−^ mice, respectively, Dsg3H1‐*Rag2* ^−/−^ T cells matured as CD4^+^ T cells in *Dsg3* ^−/−^ mice, while they were eliminated in the thymus of WT mice (Figure 2).^42^, ^84^

The central tolerance mechanism can be inconveniently disrupted. It is known that the thymus atrophies with age and acutely involutes under various inflammatory conditions.^85^, ^86^, ^87^, ^88^ It has also been reported that *Aire*‐expressing mTECs decrease along with acute thymic involution in imiquimod‐induced dermatitis. Interestingly, in this involuted thymus, Dsg3H1‐*Rag2* ^−/−^ T cells can differentiate into CD4^+^ single‐positive T cells without being eliminated.^89^ This phenomenon indicates that inflammation in peripheral tissues can affect central immune tolerance and potentially contribute to the development of autoimmune diseases. In human clinical practice, the development of PF has been reported after topical application of imiquimod cream.^90^, ^91^ Its clinical relevance has also been discussed based on the fact that imiquimod is an agonist of TLR7. In other words, TLR7 signaling is known to be activated in influenza infection, and cases of PV developed after severe influenza infection or vaccination have been described.^92^, ^93^ Although it is unknown how much thymus was involved in these cases, inflammation in the periphery seems to influence crucial tolerogenic steps that usually prevent disease development in disease‐predisposed individuals.

Even though there was no Dsg3 expression in mTEC of *Aire* ^−/−^ mice, similar to the thymic situation of *Dsg3* ^−/−^ mice, CD4^+^ T cells from *Aire* ^−/−^ mice were much less capable of inducing anti‐Dsg3 antibody production than those from *Dsg3* ^−/−^ mice, suggesting that *Aire* deficiency causes the emergence of Dsg3‐specific effector T cells in the periphery, but their pathogenicity was constrained by some mechanisms likely governed by Dsg3‐expressing peripheral tissues of *Aire* ^−/−^ mice.^83^ In addition, mice with imiquimod‐induced acute thymic involution also allow Dsg3‐specific T cell development in the thymus but do not show clinical symptoms of pemphigus, despite the expression of Dsg3 antigen in their peripheral tissues.^83^, ^89^ These facts simply suggest the existence of immune‐suppressive functions other than central tolerance, and it must be the immune tolerance mechanism operated in the peripheral tissue, peripheral tolerance.

### 5.3 Peripheral tolerance to Dsg3

The peripheral immune tolerance mechanism works in the secondary lymphoid tissues and peripheral organs, which are the sites of the immune response to pathogens invading the body. Clonal deletion and anergy induction are representative mechanisms.^94^, ^95^, ^96^ In clonal deletion, autoreactive T cells are eliminated. Anergy induction involves non‐proliferation of T cells with no reaction to antigens. A well‐known mechanism of clonal depletion is apoptosis, which occurs by allowing Fas–FasL signaling into autoreactive T cells in some cases. The mechanism of anergy is induced by insufficient CD28 signaling from antigen‐presenting cells.^94^, ^95^, ^97^ In recent years, the molecular mechanisms that inhibit T cell immune activation have also become known, involving CTLA‐4 and PD‐1 as checkpoints for immuno‐inflammation.^98^, ^99^, ^100^

The presence of anergic T cells, found in healthy individuals but rarely in patients with PV, has been reported. These cells produce IL‐10 on recognizing Dsg3‐antigen but do not proliferate (Figure 2).^101^ This observation suggests an anergy induction mechanism against Dsg3‐specific T cells. Furthermore, it has been clearly observed that there is a deletion mechanism against Dsg3‐specific T cells in mice.

**FIGURE 3:**
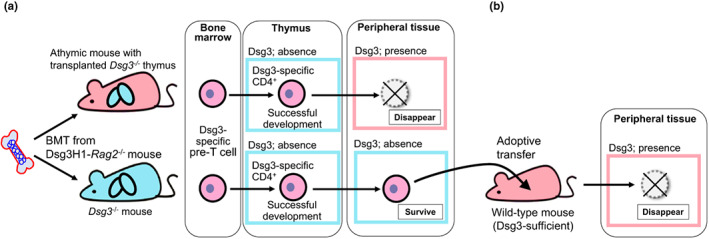
Experimental models revealing the peripheral deletion of Dsg3‐specific T cells. After bone marrow transplantation from Dsg3H1‐*Rag2* ^*−/−*^ mice into *Dsg3* ^−/−^ thymus‐transplanted athymic mice and *Dsg3* ^−/−^ mice, development of Dsg3‐specific CD4^+^ T cells is observed in thymus and skin‐draining lymph nodes. In *Dsg3* ^−/−^ thymus‐transplanted athymic mice, Dsg3‐specific CD4^+^ T cells are developed in the transplanted *Dsg3* ^−/−^ thymus and are deleted in the periphery (a, upper row). In *Dsg3* ^*−/−*^ mice, they develop in the thymus and survive in the periphery (a, bottom row). When the surviving Dsg3H1‐*Rag2* ^*−/−*^ T cells are adoptively transferred into wild‐type mice, they are deleted (b).

The mechanism of peripheral immune tolerance to Dsg3 was experimentally demonstrated at the molecular level in a mouse study using the thymus transplantation technique.^102^ The study confirmed that the peripheral immune tolerance mechanism could eliminate Dsg3‐specific CD4^+^ T cells that have developed into the periphery without central immune tolerance. Transplantation of Dsg3‐deficient thymus into athymic mice creates a chimeric condition in which Dsg3 expression is lost only in the thymus and maintained in the peripheral tissues.^84^ When bone marrow from Dsg3H1‐*Rag2* ^−/−^ mice were transplanted into the thymus transplanted mice, Dsg3H1‐*Rag2* ^−/−^ T cells were not eliminated and developed into CD4 single positive T cells in the transplanted Dsg3‐deficient thymus. Still, they were deleted in the periphery and were not detected in the lymph nodes and spleen, demonstrating the existence of peripheral immune tolerance to Dsg3 (Figure 3a).

**FIGURE 4:**
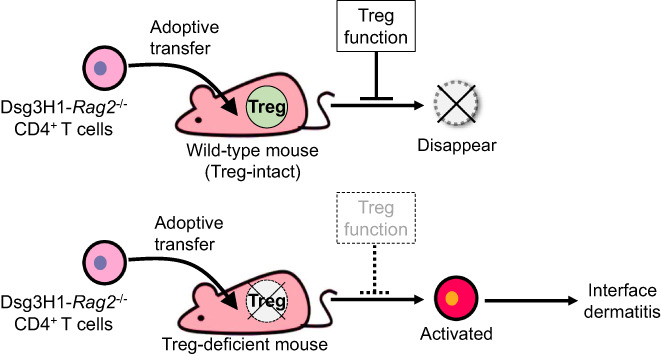
Treg‐mediated peripheral tolerance against Dsg3‐specific CD4^+^ T cells. Dsg3H1‐*Rag2* ^−/−^ CD4^+^ T cells disappear after adoptive transfer into Treg‐intact wild‐type mice. On the other hand, after transfer into Treg‐deficient mice, they are activated and induce interface dermatitis by attacking Dsg3‐expressing keratinocytes.

This peripheral deletion was also observed when Dsg3H1‐*Rag2* ^−/−^ T cells, which are developed in the thymus and distributed into the periphery in *Dsg3* ^−/−^ mice, were adoptively transferred into wild‐type mice that expressed the Dsg3 antigen in the periphery (Figure 3b). However, when Dsg3H1‐*Rag2* ^−/−^ T cells were adoptively transferred into Treg‐deficient mice, they did not disappear, infiltrated the skin, and attacked Dsg3‐expressing keratinocytes, causing interface dermatitis (Figure 4).^84^ This suggests that Tregs are essential for peripheral deletion mechanisms against Dsg3‐specific T cells.

Further analysis showed that OX40 expressed on Tregs is a key molecule required for this peripheral deletion mechanism; specifically, OX40 on Tregs contributes to the elimination of Dsg3H1‐*Rag2* ^−/−^ T cells by depriving antigen‐presenting dendritic cells of OX40L and consequently decreasing OX40 signaling in Dsg3H1‐*Rag2* ^−/−^ T cells, probably via interaction with DCs with OX40L expression lowered (Figure 2). The importance of antigen‐specific functions of Tregs can be inferred from this mechanism involving antigen‐presenting dendritic cells. Indeed, in this experimental setting, the accumulation of OX40 positive Dsg3‐specific Tregs, along with the appearance of Dsg3H1‐*Rag2* ^−/−^ T cells after adoptive transfer, has been observed using MHC class II tetramer.^84^ The study demonstrated the essential role of Tregs in removing Dsg3‐specific CD4^+^ T cells by suppressing OX40 signals as one of the crucial mechanisms in peripheral tolerance.

## 6 FUTURE PERSPECTIVE

As reviewed here, substantial knowledge of the immune responses of autoreactive T cells and their regulatory mechanisms has accumulated. To apply scientific findings from basic research to future therapeutic strategies, preclinical studies using mouse models would be very efficient and necessary, especially to elucidate detailed cellular and molecular mechanisms on the regulatory aspects of the immune system and to understand how we can prevent harmful autoimmune reactions in patients.

Future promising treatments include the use of Tregs. According to one study, eliminating autoreactive T cells by Tregs could advocate using Treg therapy, as physical elimination of autoreactive T cells is better for immunosuppression than suppressing T cell activity, leaving no chance for future reactivation of the harmful T cells. This Treg action would be effective as a treatment even after Dsg3‐specific T cells appear in the periphery and cause the disease. Furthermore, future technology handling antigen‐specific Tregs would significantly progress toward safer and more effective therapies for pemphigus and other autoimmune diseases.

## CONFLICT OF INTEREST

None declared.
